# Recurrent microdeletion at 17q12 as a cause of Mayer-Rokitansky-Kuster-Hauser (MRKH) syndrome: two case reports

**DOI:** 10.1186/1750-1172-4-25

**Published:** 2009-11-04

**Authors:** Laura Bernardini, Stefania Gimelli, Cristina Gervasini, Massimo Carella, Anwar Baban, Giada Frontino, Giancarlo Barbano, Maria Teresa Divizia, Luigi Fedele, Antonio Novelli, Frédérique Béna, Faustina Lalatta, Monica Miozzo, Bruno Dallapiccola

**Affiliations:** 1 "Casa Sollievo della Sofferenza" Hospital, IRCCS, S San Giovanni Rotondo, Italy; 2 Genetic Medicine, University Hospitals of Geneva, Geneva, Switzerland; 3 Division of Medical Genetics, San Paolo School of Medicine, University of Milan, Milan, Italy; 4 Molecular Genetics Unit, G Gaslini Children's Hospital, Genoa, Italy; 5 Cardiology Unit, Molecular Genetics Unit, G Gaslini Children's Hospital, Genoa, Italy; 6 Department of Obstetrics, Gynaecology and Neonatology, Fondazione Policlinico-Mangiagalli-Regina Elena, University of Milan, Italy; 7 Department of Nephrology, G Gaslini Children's Hospital, Genoa, Italy; 8 Clinical Genetic Unit, Department of Obstetrics and Pediatrics, University of Milan, Fondazione Policlinico-Mangiagalli-Regina Elena, University of Milan, Italy

## Abstract

**Background:**

Mayer-Rokitansky-Kuster-Hauser syndrome (MRKH) consists of congenital aplasia of the uterus and the upper part of vagina due to anomalous development of Müllerian ducts, either isolated or associated with other congenital malformations, including renal, skeletal, hearing and heart defects. This disorder has an incidence of approximately 1 in 4500 newborn girls and the aetiology is poorly understood.

**Methods and Results:**

we report on two patients affected by MRKH syndrome in which array-CGH analysis disclosed an identical deletion spanning 1.5 Mb of genomic DNA at chromosome 17q12. One patient was affected by complete absence of uterus and vagina, with bilaterally normal ovaries, while the other displayed agenesis of the upper part of vagina, right unicornuate uterus, non cavitating rudimentary left horn and bilaterally multicystic kidneys. The deletion encompassed two candidate genes, *TCF2 *and *LHX1*. Mutational screening of these genes in a selected group of 20 MRKH females without 17q12 deletion was negative.

**Conclusion:**

Deletion 17q12 is a rare albeit recurrent anomaly mediated by segmental duplications, previously reported in subjects with developmental kidney abnormalities and diabetes. The present two patients expand the clinical spectrum associated with this imbalance and suggest that this region is a candidate locus for a subset of MRKH syndrome individuals, with or without renal defects.

## Introduction

Mayer-Rokitansky-Küster-Hauser (MRKH) syndrome (MIM 277000) affects about 1 in 4500 female births, presenting with congenital aplasia of the uterus and the upper part of vagina, in association with unremarkable development of secondary sexual characteristics and normal 46, XX karyotype. This disorder can be isolated (type I; OMIM 277000) and comprises the so-called CAUV (Congenital Absence of the Uterus and Vagina) and MA (Müllerian Aplasia), or associated with renal, skeletal and/or hearing defects. Cardiac and digital anomalies are rare. Patients with associated anomalies are classified as MURCS association (MÜllerian duct aplasia, Renal Dysplasia and Cervical Somite anomalies), also indicated as MRKH type II syndrome (OMIM 601076), or GRES (Genital Renal Ear Syndrome)(OMIM 267400) if the middle ear is also affected [1]. The most common coexisting defects affect the upper urinary tract, including unilateral renal agenesis, ectopia of one or both kidneys, renal hypoplasia, horseshoe kidney and hydronephrosis [2]. As thoroughly reviewed in a recent paper [1], several inheritance models have been suggested to explain MRKH syndrome aetiology, including polygenic/multifactorial causes and non-genetic issues. Among non-genetic causes maternal diabetes and the teratogen action of thalidomide-like substances have been proposed. All the hypotheses are likely reliable, suggesting that MRKH has a wide aetiological heterogeneity [1]. The increasing number of familial cases, the pattern of congenital malformations, the co-occurrence of Müllerian aplasia in either recessive and dominant syndromes and the association with chromosome rearrangements (see [3] for a review) indicate that MRKH is a disorder occurring during embryogenesis, the genetic factors playing a crucial role. So far, *WNT4 *gene mutations have shown to be responsible for a clinically distinct subtype of this disorder, presenting with Müllerian aplasia and hyperandrogenism, with or without renal aplasia [4].

We report two patients affected by MRKH syndrome resulting from deletion of the same 1.5 Mb segment at 17q12 band, including *TCF2 *and *LHX1 *genes.

## Patients

The two patients with Müllerian aplasia reported in this study were enrolled during a multicentric study of MRKH syndrome. Twenty additional females with a clinical diagnosis of MRHK syndrome without 17q12 chromosome deletion were investigated for *TCF2 *and *LHX1 *gene mutations. All participants were informed about the study and signed a consent form approved by the Ethical Committee of each Institute involved.

### Patient 1

This patient was the second child of healthy, non consanguineous parents. The mother was 28 and the father 33 year-old at conception. Family history was unremarkable. Pregnancy was normal and delivery at term by caesarian section, because of maternal indication. Birth weight was 3,250 g (50^th ^centile), length 48 cm (10^th ^centile), and Apgar scores were 9 and 10 at 1 and 5 minutes. Growth and psychomotor development were normal, but the girl experienced several episodes of acute cystitis during infancy.

**Figure 1:**
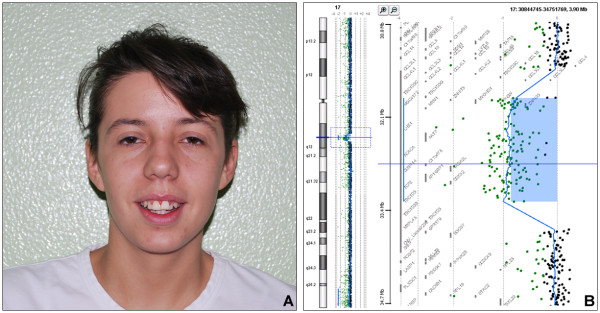
**A) Patient 1: facial appearance showing mild dysmorphisms**. B) Detail of array-CGH graphical overview of 17q12 deletion spanning about 1.5 Mb from A_16_P20635582 (31,897,238 Mb) to A_16_P20639170 (33,322,972 Mb) probes.

She was referred for a genetic opinion at age of 20 years with a diagnosis of primary amenorrhoea and abnormal genitalia. Weight was 52 kg (25-50^th ^centile), height 163 cm (50^th ^centile), and OFC 54 cm (25-50^th ^centile). She had mild dysmorphic features, including laterally sparse eyebrows, down slanted palpebral fissures, hypertricosis of upper lip (Figure 1A). A transrectal sonogram showed congenital absence of the uterus, polycystic left ovary 29 × 12 × 31 mm, and a likely left müllerian remnant 16 × 18 mm. The right ovary measured 40 × 39 × 45 mm with a functional cyst of 36 × 38 × 44 mm. A pelvic magnetic resonance imaging disclosed normal kidneys, an unusually thin bladder wall, congenital absence of uterus and vagina, bilaterally normal ovaries. Audiogram, ECG, cardiac sonogram and blood glucose level were unremarkable. Standard karyotype was normal (46, XX). At age of 20 years the proband underwent a surgical corrective procedure, with the construction of a neo-vagina. Laparoscopic exploration confirmed uterine agenesis, normal ovaries and persisting Müllerian structures. No additional anomaly or congenital defects were associated with the genital malformation.

### Patient 2

This patient was the first child of healthy non consanguineous parents. At birth, the father and mother were 30 and 29 year-old respectively. A younger sister was normal. Family history was negative for renal, genital or skeletal defects. Pregnancy was unremarkable until the 7^th ^month, when US scan disclosed bilateral foetal renal cysts. Chromosome analysis on amniocytes was normal (46, XX). The baby was born at term by caesarean section for obstetric reasons. Birth weight was 3,530 g (50^th ^centile) and length 51 cm (75^th ^centile).

The patient came to our observation for the first time at the age of 10 months for evaluation of renal cysts and then revaluated periodically in the subsequent years. Renal ultrasound at age of 5 years showed bilaterally small-sized multicystic kidneys. Both kidneys had diffuse parenchymal hyperecogenicity, poor cortico-medullary differentiation, and small subcapsular cysts. The collecting system was not dilated and urinary bladder wall was regular. These changes were confirmed in subsequent ultrasounds. The developmental milestones and psychomotor development were normal. At the age of 12 years, menarche was complicated by haematocolpos due to agenesis of the upper and middle thirds of vagina, which were surgically corrected. Laparoscopy demonstrated Müllerian malformations with right unicornuate uterus, no cavitating rudimentary left horn, right hematosalpinx and surgically corrected agenesis of the upper and middle thirds of vagina. Ophtalmologic examination, ECG, 24 hour holter measurement, renal function test, liver function test, parathyroid hormone measurement, blood glucose level, uric acid level, blood protein level, 24 hour proteinuria and oxaluria were unremarkable.

At age of 15 years weight was 62.5 Kg (75-90^th ^centile), height 168 cm (75-90^th ^centile), and OFC 55.8 cm (50-98^th ^centile).

## Methods

### Array-CGH

Genomic oligonucleotide-array (244 K; Agilent Technologies Inc., Palo Alto, CA) was used according to standard protocol with some modifications. For each patient genomic DNA was extracted from 2 ml of an EDTA blood sample. Patients' DNA and a female reference DNA (Promega, Madison, WI) were Alu I and Rsa I double-digested (Promega) and purified using DNA Clean and Concentrator Kit (Zymo Research Corporation; Orange; CA). Digested DNA was quantified by the fluorimeter (BioRad, Hercules, CA) and 1 μg of each patient and reference DNA were labelled with Cy5-dCTP and Cy3-dCTP (Agilent Technologies Inc.). The Cy5 and Cy3 labelled DNAs were combined and cleaned-up using Micron YM-30 columns (Millipore, Bedford, MA). After the addition of blocking agents and the hybridisation buffer (Agilent Technologies, Palo Alto, CA), DNAs were denaturated at 95°C and hybridised at 65°C for 40 hours with rotation. After washes, dried slides were immediately scanned on the Agilent scanner, imaged with Feature Extraction software (v8.5.3) and analysed using the DNA analytics software (v4.0) (Agilent Technologies Inc.).

A chromosome 17 custom-microarray was designed *in silico *using the web Agilent eArray database version 4.5 , in order to test other MRKH patients for the 17q12 deletion and to eventually compare the size of the rearrangements. All the Agilent's high-density CGH probes mapping to chromosome 17 were included in the array format 8 × 15 K. The Agilent-optimized chromosome X probes have been used as control (March 2006 release).

### FISH

Deletions were confirmed by FISH analysis using RP11-19G24 clone selected from the 32 K genomic library (BACPAC Resources Center; ), based on mapping data (UCSC; ., March 2006 release). DNA was extracted by a Quantum Prep MiniPrep Kit (BioRad, Hercules, CA) and SpectrumGreen-dUTP or SpectrumOrange-dUTP labeled using the Nick Translation kit (Vysis Inc., Downers Grove, IL) according to the manufacturer's protocol. Metaphase spreads were obtained following standard procedures and FISH analysis was performed as previously described [5].

### Mutational Analysis

To amplify the entire coding sequence and flanking intronic portions of *TCF2 *and *LHX1*, 9 and 5 primer pairs were designed respectively. The PCR reactions (except *LHX1 *exon 2) were performed in a 25 μl volume containing 1.5 mM MgCl_2_, 250 μM dNTPs, 1 μM of each primer, 50 ng of genomic DNA and 1.5 U of AmpliTaq Gold DNA polymerase (Applied Biosystem, Foster City, CA) with an initial denaturation step of 12 min at 95°C followed by 35 cycles at 94°C, 30 sec; 60°C, 30 sec; 72°C, 30 sec and a final elongation of 7 min at 72°C. *LHX1 *exon 2 (high GC content) has been amplified in a 25 μl volume containing 1 mM MgSO4, 0.6× PCR Enhancer Solution, 250 μM dNTPs, 1 μM of each primer, 50 ng of genomic DNA and 1.5 U of Platinum Pfx DNA Polymerase (Invitrogen, Carlsbad, CA). PCR products were purified using ExoSAP-IT (USB Corporation, Cleveland, OH) and sequenced directly on an automated sequencer (ABI 3100; Applied Biosystem) using the ABI-PRISM big-dye Terminator Cycle Sequencing Ready Reaction kit (Applied Biosystem).

## Results

Array-CGH analysis performed on patients 1 and 2 disclosed a *de novo *deletion at 17q12 band (Figure 1B) confirmed by FISH using a specific BAC clone. FISH analysis extended to parents showed a normal hybridization pattern, demonstrating the *de novo *origin of the rearrangement. In both cases the deletion spanned about 1.5 Mb from A_16_P20635582 to A_16_P20639170 (31.897.238-33.322.972 Mb) probes (UCSC Genome Browser; , March 2006 Release), including16 RefSeq genes, and it was not listed among the copy number polymorphisms (Database of Genomic Variants; ). Subsequently, 20 consecutive patients with a clinical diagnosis of MRKH syndrome were tested by a focused chromosome 17 array-CGH analysis in order to assess the prevalence of 17q12 deletion in females affected by MRKH. This analysis was negative. These 20 patients were investigated also by direct sequencing of candidate genes mapping to 17q12 (*TCF2 *and *LHX1*). Molecular testing disclosed a number of known polymorphisms, but no pathogenic mutation (Table 1).

**Table 1:** Mutational analysis of candidate-genes

***Patient ID***	***TCF2***	***LHX1***
RK104		

RK105	1654-21 C/T	

RK106		841+26 A/G

RK107		841+26 A/G

RK111	1654-21 C/T	NO DNA

RK112	1653+48insC; 1654-21 C/T	

RK113	1654-21 T/T	

RK114	1654-21 C/T	

RK115		170+42 G/T

RK116	1654-21 C/T	

RK117		

RK118		676-34 C/T; 841+26 A/G

RK119		841+26 A/G

RK120	1654-21 C/T	

RK121	1653+48insC	

RK122	1654-21 T/T	

RK123	1654-21 C/T	

RK124		

RK125	1653+48insC	

RK126		

List of the known polymorphisms disclosed by the mutational analysis of *LHX1 *and *TCF2 *genes in 20 selected chromosomally normal MRKH females, tested negative for 17q12 deletion.

## Discussion

An increasing number of familial cases suggest that MRKH syndrome can be inherited as an autosomal dominant incompletely penetrant trait, either due to single gene mutation or chromosomal imbalances [1]. Clinical features are consistent with a developmental defect attributable to an initial affection of the intermediate mesoderm leading to an alteration of the blastema of the cervicothoracic somites and the pronephric ducts [6], but developmental genes investigated, such as *WT1*, *HOXA7*, *HOXA13 *and *PBX1*, did not reveal any pathogenic mutation [7,8]. Among chromosome causes, an identical t(12;14)(q14;q31) detected in two unrelated Indian females, a maternally inherited terminal deletion of 4q and 22q11.21 deletion, overlapping the DiGeorge syndrome region, have been described in females with syndromic MRKH. However, so far no candidate gene was identified in the unbalanced regions [9-11].

Array-CGH technique has offered in the last years new opportunities to discover cryptic chromosome imbalances causative of congenital malformations. This approach allowed Cheroki et al. to confirm the involvement of 22q region in the MRKH aetiology [12] and to detect a *de novo *deletion at 17q12 in one patient presenting with absent uterus, severe learning disability and seizures, without diabetes and renal malformations [13]. We have reported two patients evaluated for Müllerian ducts aplasia in which array-CGH analysis disclosed an identical deletion at chromosome 17q12, spanning about 1.5 Mb of genomic DNA. One patient displayed congenital absence of uterus and vagina, with bilaterally normal ovaries without any additional anomaly, while the other had agenesis of the upper part of vagina, right unicornuate uterus and non cavitating rudimentary left horn associated with bilaterally multicystic kidneys. In both patients psychomotor development was normal. This is a rare albeit recurrent imbalance detected so far in a few other subjects investigated using genomic microarray. Mefford et al. identified the same 1.5 Mb deletion in a foetus presenting with grossly abnormal, dysplastic multicystic kidneys and compared this case with five paediatric patients with renal disease and three subjects affected by isolated MODY5. It was found that the breakpoints were identical in all but one case, who displayed a larger deletion, and occurred at segmental-duplication clusters with multiple regions of high identity (up to 99%) [14]. The deleted segment encompassed 16 known genes, including *TCF2 *and *LHX1*. Mutations in *TCF2 *gene, also known as hepatocyte nuclear factor-1-beta (*HNF1β*), which occur in individuals affected by maturity-onset diabetes of the young type 5 (MODY5; MIM 137920) and renal manifestations [15], have been associated with Müllerian disorders [1]. In particular, two of four affected females in a MODY5 family segregating *TCF2 *mutations had Müllerian aplasia [16], while in another MODY5 family, the proband displayed cystic kidneys and uterus didelphys and her affected second son had renal cysts and hypospadias [17]. *TCF2 *is expressed in renal metanephroi at preglomerular stages during metanephrogenesis [18]. Urinary and genital system are embryologically correlated, both originating from a common mesodermal ridge. Edghill et al. [19] have estimated that about 10% of subjects with *TCF2 *mutations also display some genital system anomaly. *LHX1 *encodes a transcription factor with a DNA-binding homeodomain and two cysteine-rich LIM domains that are thought to be involved in protein-protein interactions [20]. In mouse, this gene was shown to be involved in genitourinary system developmental processes, including Müllerian ducts [21]. To assess the contribution of these genes to MRKH syndrome, we performed a mutational analysis of 20 non deleted MRKH females. Analysis of 40 chromosomes disclosed several polymorphic changes, in the absence of any pathogenic variation.

## Conclusion

The recurrent 17q12 deletion is associated with a wide clinical spectrum which is further expanded by the patients reported in this study. Indeed, 17q12 deletion appears to be pathogenetically related with a subset of individuals affected by MRKH syndrome, with or without renal disease. The present data also recommend searching for 17q12 hemizygosity and/or mutations in the candidate *TCF2 *and *LHX1 *genes, both in type I and type II MRKH syndrome.

## Competing interests

The authors declare that they have no competing interests.

## Authors' contributions

This paper reports the results of a multicenter study: clinical evaluation of patients, including dysmorphologic, cardiac, urogenital and gynecologic examination, has been performed by AB, GF, GB, MTD, LF and MM; LB drafted the manuscript and participated in microarray analysis; SG, CG, MC, AN and FB performed cytogenetic and molecular studies; FL and BD critically revised the manuscript and have given final approval of the version to be published. All authors read and approved the final manuscript.

## Consent

Written informed consent was obtained from the patient for publication of the accompanying image.
